# Comparison of the Structure and Activity of Glycosylated and Aglycosylated Human Carboxylesterase 1

**DOI:** 10.1371/journal.pone.0143919

**Published:** 2015-12-11

**Authors:** Victoria Arena de Souza, David J. Scott, Joanne E. Nettleship, Nahid Rahman, Michael H. Charlton, Martin A. Walsh, Raymond J. Owens

**Affiliations:** 1 UK OPPF-UK, The Research Complex at Harwell, Rutherford Appleton Laboratory Harwell Oxford, Oxfordshire, United Kingdom; 2 Division of Structural Biology, Henry Wellcome Building for Genomic Medicine, University of Oxford, Roosevelt Drive, Oxford, United Kingdom; 3 The Research Complex at Harwell, Rutherford Appleton Laboratory Harwell Oxford, Oxfordshire, United Kingdom; 4 School of Biosciences, University of Nottingham, Sutton Bonington Campus, Sutton Bonington, Leicestershire, United Kingdom; 5 Chroma Therapeutics Ltd., 93 Innovation Drive Milton Park, Abingdon, United Kingdom; 6 Diamond Light Source, Harwell Science and Innovation Campus, Didcot, United Kingdom; Griffith University, AUSTRALIA

## Abstract

Human Carboxylesterase 1 (hCES1) is the key liver microsomal enzyme responsible for detoxification and metabolism of a variety of clinical drugs. To analyse the role of the single N-linked glycan on the structure and activity of the enzyme, authentically glycosylated and aglycosylated hCES1, generated by mutating asparagine 79 to glutamine, were produced in human embryonic kidney cells. Purified enzymes were shown to be predominantly trimeric in solution by analytical ultracentrifugation. The purified aglycosylated enzyme was found to be more active than glycosylated hCES1 and analysis of enzyme kinetics revealed that both enzymes exhibit positive cooperativity. Crystal structures of hCES1 a catalytically inactive mutant (S221A) and the aglycosylated enzyme were determined in the absence of any ligand or substrate to high resolutions (1.86 Å, 1.48 Å and 2.01 Å, respectively). Superposition of all three structures showed only minor conformational differences with a root mean square deviations of around 0.5 Å over all Cα positions. Comparison of the active sites of these un-liganded enzymes with the structures of hCES1-ligand complexes showed that side-chains of the catalytic triad were pre-disposed for substrate binding. Overall the results indicate that preventing N-glycosylation of hCES1 does not significantly affect the structure or activity of the enzyme.

## Introduction

Carboxylesterases are a family of enzymes that act on a variety of both exogenous (e.g. cocaine, heroin) and endogenous (e.g. acyl-CoA esters) substrates. They are defined by their ability to hydrolyze ester, amide, or thioester bonds to their corresponding alcohol, amine or thiol and free acid in a diverse range of chemically distinct compounds [1]. Genes coding for five carboxylesterases have been identified in the human genome (hCES1-5) [2], with CES 1, 2 and 3 appearing to be the functionally significant enzymes. All three enzymes show differential tissue expression, with cells of the monocyte/macrophage lineage being the principal source of hCES1 outside hepatocytes [3]. hCES1 and 2 are localised to the endoplasmic reticulum (ER) via the KDEL receptor. The enzymes hydrolyze substrates via a two-step ping pong mechanism that includes the formation and degradation of an acyl-enzyme intermediate, using water as a transitional nucleophile [4]. Intense interest in these enzymes stems from their critical role in Phase 1 metabolism and activation of pro-drugs, notably the anti-cancer agent, CPT-11[5].

In 2003, the first crystal structures of hCES1 in complex with narcotic analogues [6] were reported, showing that the enzyme forms a hexamer from trimers. Over the past 10 years, many more structures of hCES1 have been determined with the highest resolution structure reported to date at 2.0 Å (2h7c [7]). Carboxylesterases comprise three distinct domains; a central domain containing the catalytic triad (S221, H467 and E354), a regulatory domain (RD) and an α/β domain. The active site occupies a 10-15Å deep hydrophobic pocket at the interface of the three domains with all three catalytic residues arranged such that a proton transfer chain can be established [6]. It also contains the C-terminal helix of the enzyme. The RD is mainly helical; containing two disordered loops and has been proposed to regulate substrate binding and product release [8]. Published structures show that the RD of the enzyme exhibits high thermal displacement parameters, indicating dynamic mobility within this region [6–8]. The αβ domain or hydrolase fold, lies adjacent to both the catalytic and regulatory domain. This domain is common to a number of hydrolytic enzymes of differing catalytic functions and phylogenetic origin [9]. Examination of enzyme: substrate complexes has revealed the presence of two non-selective substrate binding sites in addition to the catalytic site. The ‘Z-site’ which is located within the regulatory domain of the enzyme [6,10], and that is proposed to control the trimer–hexamer equilibrium of the enzyme and a ‘side-door’ secondary pore that leads into the active site from the surface of the enzyme.

hCES1 has a single N-linked glycosylation site at N79 which is conserved at the equivalent position in orthologues from other species (www.uniprot.org). Over 20 years ago, Kroetz *et al*. provided evidence that N-linked glycosylation was essential for maximal catalytic activity in hCES1 for simple aromatic and aliphatic esters [11]. The enzyme was expressed in insect cells using the baculovirus system with the addition of tunicamycin in the culture media to inhibit GlcNAc phosphotransferase (GPT) and hence produce non-glycosylated enzyme. In addition, a structural role for terminal sialic acids of N-glycosylated hCES1 in stabilising the trimeric enzyme has been proposed [9,10], though as an ER resident, the enzyme would not normally be sialylated. Nevertheless, a role for glycosylation in the activity of hCES1 has become an established fact in the scientific literature [12]. However some early work on human triacylglycerol hydrolase appears to have been overlooked. This microsomal enzyme which hydrolyses cytoplasmic triacylglycerol is an isoform of hCES1 and it has been shown that the point mutation, N79A, does not affect activity of the enzyme that was also produced in insect cells [13].

To provide a definitive insight into the importance of N-glycosylation on the structure and activity of human hCES1, the crystal structure of the enzyme with the mutation, N79Q, has been determined. The structure-function relationship of this aglycosylated hCES1 was explored alongside the wild type enzyme and a catalytically inactive mutant (S221A).

## Methods

### Protein production

DNAs encoding hCES1 and hCES1 S221A optimized for combined human and insect codon usage were ordered from GeneArt® (Life Technologies). The hCES1 N79Q mutation was introduced into hCES1 by PCR strand overlap extension [14]. Genes were inserted into the mammalian cell expression vector, pOPINTTGneo [15], by Infusion cloning as described previously [16]. For the production of hCES1 and hCES1 S221A, stable HEK293 Gnt1^-^/^-^ [17] cell lines were generated by selection with G418. hCES1 N79Q was produced by transient expression in HEK 293T cells (ATCC® CRL-11268™) [18]. Secreted proteins were purified using an automated protocol consisting of nickel affinity chromatography using a HiScreen™ Ni FF (GE Healthcare) column followed by size exclusion chromatography using a HiLoad 16/60Superdex 200 column (GE Healthcare) on an ÄKTAxpress unit [19]. Purified proteins were de-glycosylated by incubating with PNGase F (Sigma-Aldrich) overnight at 37 ^0^ C. Samples were analysed by SDS-polyacrylamide gel electrophoresis under reducing conditions and gels stained using InstantBlue™ (Expedeon).

### Analytical ultracentrifugation

Sedimentation velocity experiments were carried out on a Beckman XL-I analytical ultracentrifuge (Beckman-Coulter). Sedimentation velocity was performed at 20°C using 2 channel centre pieces, with protein loading concentrations of 2 mg/ mL, 1.0 mg/ mL and 0.5 mg/ mL in 20 mM TrisHCl, pH 7.5 containing 200 mM NaCl. Data were obtained at 40,000 rpm, using a Beckman 50Ti rotor, with the cells scanned radially with interference optics and with absorbance optics at a wavelength of 280 nm. Scans were obtained every 10 minutes and data were analyzed using the program SEDFIT v11.3 (www.analyticalultracentrifugation.com). Sedimentation coefficient distributions were obtained using the c(S) methodology114, and figures were created in GUSSI 1.0.3. Solution densities and viscosities were measured directly using an Anton Paar DMA5000 densitometer/viscometer.

### Enzyme kinetics

Esterase activity was measured using 4-nitrophenyl acetate (4-NPA) as the substrate [20][20] at varying concentrations (0–3000 μM). The rate of hydrolysis of 4-NPA was followed by measuring the production of the nitrophenolate anion at 405 nm (ε 405 = 18000 M ^-1^ cm ^-1^) in a Paradigm Plate Reader (Beckman Coulter). The assay was carried out at 37°C with shaking and A_405_ readings taken at 30 second intervals over 30 minutes. Experiments were carried out in triplicate, results averaged and the amount of product formed plotted against time. Rates of reaction at different substrate concentrations were calculated from the linear part of the curves. All *r* ^2^ values exceeded 0.99. An allosteric sigmoidal substrate-velocity model (nonlinear regression) was fitted to the data using GraphPad Prism version 6 (www.graphpad.com) and kinetic parameters calculated using the following equation:

$$Y=\frac{V_{max}x^{h}}{\left(K_{half}^{h}+x^{h}\right)}$$

Where *Y* = initial velocity, *x* = concentration of substrate, *V*_*max*_ is the maximum velocity of the enzyme, *h* = Hill coefficient and *K*_*half*_ is the concentration of substrate that produces a half-maximal enzyme reaction rate.

### Crystallization and structure solution

hCES1 and hCES1 S221A were concentrated to 5 mg/ml and hCES1 N79Q to 6 mg/ml in 20 mM Tris pH 7.5, 200 mM NaCl and crystallization trials set up in 200 nL (100nL protein plus 100 nL reservoir solution) drops in a 96 well format [21][21]. hCES1 crystals were grown at 20°C in 0.1 M MES/imidazole, pH 6.5 containing 0.03 M each of diethylene glycol, triethylene glycol, tetraethylene glycol, pentaethylene glycol, 10% (w/v) polyethylene glycol 4000, 20% (w/v) glycerol. Both hCES1 S221A and hCES1 N79Q were crystallized at 4°C in respectively, 0.1 M Bicine/Trizma® base, pH 8.5, containing 0.03 M each of diethylene glycol, triethylene glycol, tetraethylene glycol, pentaethylene glycol, 10% (w/v) PEG 4000, 20% (v/v) glycerol and 0.1 M Bis-Tris propane, pH 7.5, containing 20% (w/v) polyethylene glycol 3350, 0.2 M NaI. For crystals that were harvested from conditions optimised from the Morpheus**®** screen [22] (hCES1 and hCES1 S221A), no cryo-protection was needed. For the hCES1 N79Q crystal grown from conditions identified from the PACT screen [23], 25% glycerol was added directly as a cryo-protectant. Diffraction data were collected at Diamond Light Source (DLS), Oxfordshire, on beamlines I03 and I04. The structure of hCES1 was solved by molecular replacement with PHASER [24] using the coordinates of the human carboxylesterase PDB entry 2h7c as a search model [7]. Phases calculated from this initial model were used for manual completion of the structure using COOT [25] with iterative cycles of refinement with REFMAC [26]. This high resolution structure of hCES1 was then used as the search model for molecular replacement of the other two structures presented here. For the high resolution structure of hCES1 S221A where data extended to 1.48 Å, anisotropic refinement of individual atomic displacement parameters was feasible. MolProbity4 was used for structure validation. Diffraction data and refinement statistics are shown in Table 1. SHP was used for structural comparisons [27] and images for figures prepared with PyMOL (http://pymol.sourceforge.net/). The coordinates and structural factors for hCES1, hCES1 S221A and hCES1 N79Q have been deposited in the Protein Data Bank under accession numbers 5a7f, 5h7g and 5a7h, respectively.

**Table 1:** Data collection and refinement statistics.

	hCES1 wild type	hCES1 S221A	hCES1 N79Q
Data Collection			
X-Ray Source	DLS, I04	DLS, I03	DLS, I04
Wavelength (Å)	0.9795	0.97625	0.9795
Space Group	R3: H	R3: H	R3: H
Unit Cell Parameters			
a, b, c [Å]	114.72, 114.72, 117.78	115.39, 115.39, 128.14	115.47, 115.47, 127.28
α, β, γ [°]	90, 90, 120	90, 90, 120	90, 90, 120
Resolution range [Å]	50.00–1.86 (1.88–1.86)	53.94–1.48 (1.52–1.48)	78.63–2.01 (2.06–2.01)
Completeness (%)	97.0 (71.5)^a^	98.4 (91.8)	98.3 (98.0)
Rmerge^b^ (%)	6.5 (43.3)	4.7 (69)	6.3 (61)
CC1/2^c^ (High resolution shell)	0.785	0.612	0.64
<I>/σ(<I>)	17.4 (1.8)	11.1 (1.0)	9.7 (1.3)
Multiplicity	2.8 (2.3)	2.9 (2.0)	3.0 (3.0)
Data Refinement			
No. of reflections	132,450	303,901	125,184
No. of unique reflections	46, 879	104,388	41,414
R-factor^d^(%)	15.83	12.96	17.9
Rfree^e^(%)	18.76	17.19	22.16
R.m.s. deviations			
Bond lengths (Å)	0.013	0.012	0.012
Bond angles (°)	1.63	1.51	1.4694
Wilson B-factor (Å2)	21	19.4	19.2
Mean B-factor (Å2)	18.7	31	43.37

^a^Values in parentheses refer to data in the highest resolution shell for each protein.

^b^R_merge_ = ∑_*hkl*_ ∑_*j*_|*I*_*hkl*,*j*_ − 〈*I*_*hkl*_〉|/∑_*hkl*_ ∑_*j*_ *I*_*hkl*,*j*_.

^c^CC values are the half-set correlation coefficients as described by Karplus & Diederichs [49]

^d^R-factor = ||*F*_*obs*_| − |*F*_*calc*_||/∑|*F*_*obs*_|;

^e^R_free_ = R-factor for a selected subset (5%) of the reflections that were not included in prior refinement calculation.

## Results and Discussion

### Production and biophysical characterisation of glycosylated and aglycosylated human HCES1

**Fig 1:**
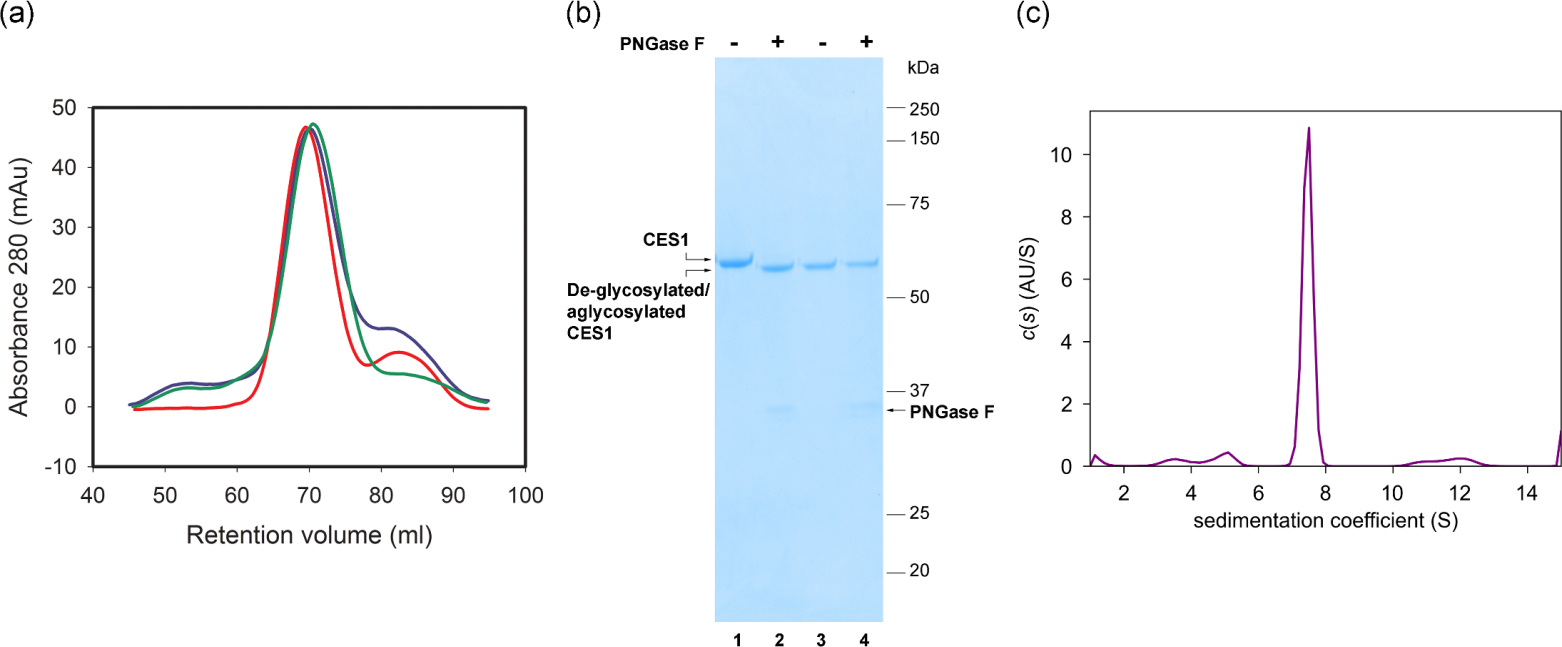
Purification of human carboxylesterases. (a) Size exclusion profiles of purified hCES1 (blue trace) hCES1 N79Q (green trace) and hCES1 S221A (red trace) enzymes from media of transfected HEK cells. Samples were run on a HiLoad 16/60Superdex 200 column (GE Healthcare) in 200 mM NaCl, 20 mM Tris-HCl, pH 7.5. The peak corresponds to a molecular weight of approximately 160 kDa as estimated from the elution volumes of globular proteins of known molecular weight: Aprotinin (6.5 kDa) Ribonuclease A (13.7 kDa) Carbonic Anhydrase (29 kDa), Ovalbumin (44 kDa), Conalbumin (75 kDa) Aldolase (158 kDa) Ferritin (440 kDa) and Blue Dextran 2000. (b) SDS-polyacrylamide gel of purified CES1 (lanes 1 and 2) and CES1 N79Q (lanes 3 and 4) untreated (lanes 1 and 3) and treated with PNGaseF (lanes 2 and 4). (c) The sedimentation velocity distribution for hCES1 N79Q. Data for hCES1 and hCES1 S221A gave the same profiles.

Recombinant hCES1 proteins for structural studies have been produced using the *Spodoptera frugiperda* (Sf21) [28] insect expression system, infecting the cells with a baculovirus and purifying the enzyme from the supernatant [29–37]. The yields of purified enzymes were reported as 7.5–12.5 mg/ L cell culture [38]. As an alternative to production in insect cells in culture, Greenblatt *et al*., reported using *Trichoplusia ni* (whole cabbage looper) larvae with a yield of 9 mg protein from ~ 1 kg of infected caterpillar larvae [39]. Here, recombinant hCES1 enzymes were produced in HEK293 cells using either transient expression (hCES1 N79Q) or from stable cell lines (hCES1 and S221A). The ER retention motif, (HIEL) was deleted from the sequences so that proteins were secreted into the cell media and recovered by metal chelate chromatography via a C-terminal histidine tag added to the sequences. In order to match the high mannose glycoforms that would be present on the native enzyme, transient expression in HEK293T cells was carried out in the presence of the mannosidase inhibitor kifunensine [40]. The HEK Gnt1^-^/^-^ mutant cell line in which N-glycosylation is restricted to GlcNAc_2_Man_5_ was used for generating stable cell lines. The yields of the aglycosylated hCES1 N79Q, glycosylated hCES1 and S221A enzymes were 39 mg/L, 64 mg/L and 68 mg/L respectively showing that N-glycosylation is not required for expression and secretion of the enzyme consistent with previous results [11,13]. The calculated monomeric molecular weight of hCES1 is 60.9 kDa (excluding N-glycosylation) and on size exclusion chromatography the three proteins eluted at around 160 kDa consistent with assembly into trimers (Fig 1A). To assess the N-glycosylation states of the purified proteins, samples were treated with PNGase F and analysed by SDS-polyacrylamide gel electrophoresis. As shown in Fig 1B, CES1 migrated as single species above CES1 N79Q consistent with full occupancy of the N79 glycosylation site. Following, de-glycosylation with PNGase F, the CES1 co-migrated with the N79Q aglycosylated protein confirming removal of the single N-glycan. As expected, the mobility of the N79Q protein was not altered by PNGase treatment (Fig 1B).

The oligomeric states of the hCES1 and N79Q proteins were investigated by analytical ultracentrifugation in sedimentation velocity experiments. The results showed that all three forms of hCES1 were mainly trimeric with an apparent molecular weight of 152 kDa (Fig 1C). A small amount of monomer was also identified (64 kDa), as well as some aggregation at a higher molecular weight. The corrected sedimentation coefficient ($S_{20,w}^{0}$) of the trimer was calculated to be 7.5 *S*, and the frictional ratio (*f*/*f*_o_) was 1.52. Together the results confirm that hCES1 behaves overwhelmingly as a trimer in solution with no evidence for significant hexamer formation and that glycosylation plays no role in trimer assembly.

### Analysis of enzyme activity

**Fig 2:**
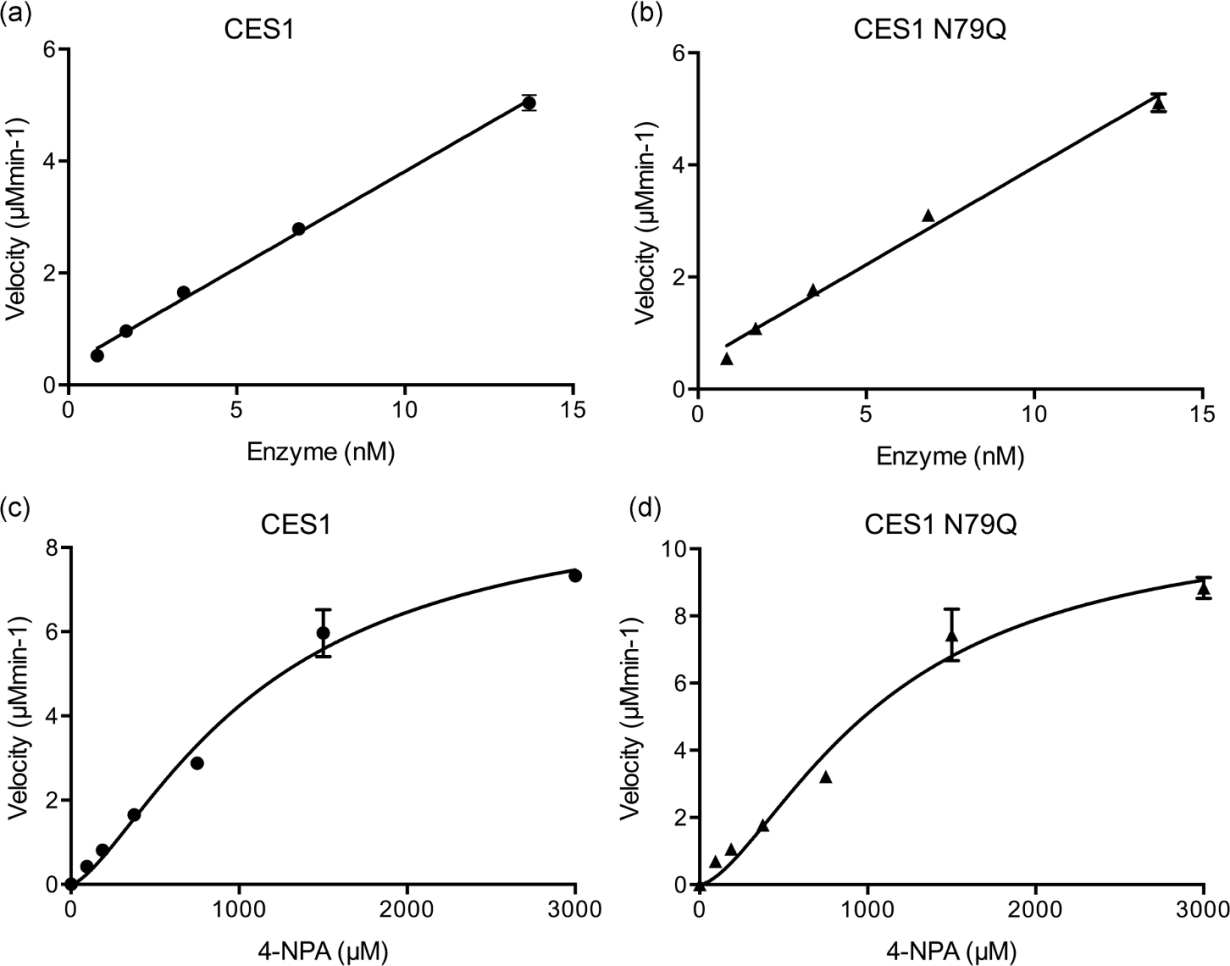
Enzyme activity of human carboxylesterases. Plots of initial rates of reaction against enzyme concentration assayed as described in the Methods section at a substrate concentration of 750 μM 4-NPA (a) CES1 (b) CES1 N79Q. Plots of initial reaction rates against substrate concentration for the hydrolysis of 4-NPA by (c) hCES1 and (d) hCES1 N79Q (3.4 nM each enzyme). The molarity of the enzyme was calculated assuming 100% trimer with a molecular weight of 182.7 kDa.

hCES1 native and N79Q hydrolysed the substrate 4-nitrophenyl acetate (4-NPA) demonstrating their functional enzymatic activity; as expected the S221A mutant was inactive. Activity increased linearly with enzyme concentration (Fig 2A and 2B) and the specific activities for hCES1 and for hCES1 N79Q were 73 µmol min^-1^ μg^-1^, 98 µmol min^-1^ μg^-1^ respectively. Glycosylation does not appear to markedly affect activity consistent with other reports for recombinant hCES1 [41].

In contrast to previous results, e.g. [41–43], the activity of both enzymes did not follow classical Michaelis-Menten kinetics. Initial reaction velocities (*V*_*0*_) plotted against substrate concentration (*S*) for both hCES1 and the N79Q mutant gave sigmoidal rather than hyperbolic profiles indicative of positive cooperativity (Fig 2C and 2D). Fitting the data to an allosteric model by non-linear regression gave values for the Hill coefficient (*h*) for both enzymes >1.00 (Table 2). It is interesting to note that native pig and human liver microsomal carboxylesterases have previously been reported to exhibit positive cooperativity [44,45]. This phenomenon is typical of enzymes comprising more than one identical subunit and can be interpreted by reference to one of two models namely the Monod-Wyman-Changeux (MWC) concerted model [46] and the Koshland-Nemethy-Filmer (KNF) sequential model [47]. Both models postulate that each subunit can exist in two different conformational states nominally termed R (relaxed) and T (tense) but differ in their assumptions about subunit interaction and the pre-existence of both states. In the MWC model, all subunits are in presumed to be in the same conformation with T and R states in equilibrium in the absence of substrate [46]. One state is assumed to have a higher binding affinity for the substrate than the other such that on binding of a ligand (substrate or effector) the equilibrium is shifted in favour of the more active state. The KNF model postulates that substrate binding involves a process of induced fitting and that this conformational change affects other active sites such that the protein transitions from one state into a more active one. However, unlike in the MWC model, each subunit can undergo conformational change independently. It is not *a priori* possible to distinguish between these two models to explain the behaviour of CES1, though the observation that substrate binding does not appear to be associated with a conformational change in the active site would favour the concerted model. It has been suggested that the so-called Z-site, shown to be occupied by substrates in some hCES1-ligand complexes is an allosteric binding site [6]. Thus transitions from low to high substrate binding affinity may be modulated by substrate binding to a non-catalytic site on the enzyme. Most interestingly, analysis of the kinetics of natural pig microsomal carboxylesterase 1 according to a concerted model of cooperativity suggested that each subunit of the trimeric enzyme had two substrate binding sites [45]. Overall the enzyme assay results confirm that N-glycosylation does not affect binding of the 4-NPA substrate and are consistent with recent results for the hCES1 expressed in *E*.*coli* as an insoluble protein and subsequently refolded *in vitro* [41]. Analysis of enzyme kinetics show that recombinant hCES1 exhibits positive cooperativity, though the molecular mechanism for this remains unknown.

**Table 2:** Enzyme kinetic data.

Parameter	CES1	CES1 N79Q
Vmax (μMmin^-1^)	9.25 +/- 1.30	10.92 +/- 2.01
h	1.46 +/- 0.26	1.57 +/- 0.4
Khalf (μM)	1123 +/- 280.2	1090 +/- 343.3
Goodness of Fit (r^2^)	0.9915	0.9819

Values are given +/- standard errors from the analysis of *V0* vs *S* using the allosteric model in GraphPad Prism version 6

### Comparison of the structures of glycosylated and aglycosylated human hCES1

**Fig 3:**
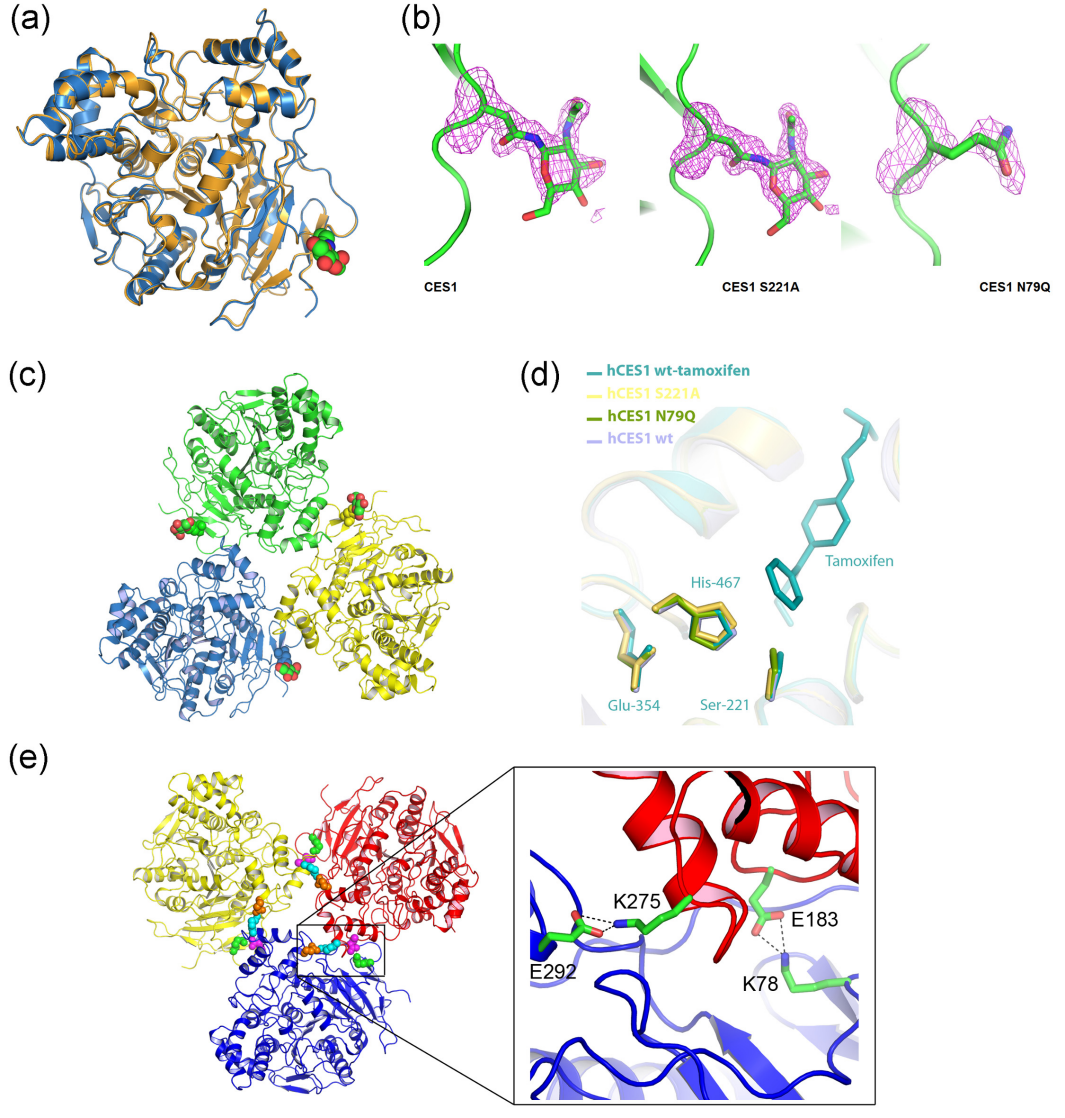
Crystal structures of human carboxylesterases. (a) Overlay of hCES1 (blue) and hCES1 N79Q (gold) (b) Views of the glycosylation site for hCES1, S122A hCES1 and the N79Q mutant. The *F*o–*F*c omit map electron density is shown carved around the glycosylation site (N79) and contoured at a level of 3 σ (c) Trimer of hCES1 generated by the space group symmetry, N-acetylglucosamine sugars attached to N97 of hCES1 are represented as spheres in red and green (d) Superpostion of the catalytic triad of hCES1 (mauve), hCES1 N79Q (green), hCES1 S221A (yellow) hCES1 tamoxifen complex (PDB id 1ya4, turquoise). Residues are in stick representation. The bound tamoxifen is coloured turquoise. (e) Cartoon representation of the hCES1 trimer generated by the space group symmetry with the K78:E183 and K275:E292 salt bridges shown in spheres (K78 in green, E183 in magenta, K275 in cyan and E292 in orange).

All three proteins readily crystallized in the space group, R3: H, a new space group for hCES1 which has most commonly crystallized in the orthorhombic space group *P*2_1_2_1_2_1_. The structures of hCES1, S221A and N79Q mutants were solved by molecular replacement using hCES1 in complex with Coenzyme A determined to 2.0 Å resolution (PDB code: 2h7c) as the initial search model and refined to 1.86 Å, 1.48 Å and 2.01 Å respectively (Table 1). These represent the highest resolution structures of hCES1 solved to date in the absence of any substrates. In all three structures, the asymmetric unit contained one monomer of hCES1, containing, two disulphide bridges were present, C87- C116 and C274—C285 as seen in other hCES1 structures. Superposition of the monomeric structures showed no differences between the glycosylated and aglycosylated hCES1 (Fig 3A). The individual monomers superimposed with root mean square deviations (rmsd) of 0.47 Å (hCES1) and 0.3 Å (S221A) for all C_α_ atoms (Table 3). In the crystal structure of hCES1 and hCES1 S221A, an N-acetylglucosamine adduct was observed attached to the side chain of residue N79. (Fig 3B). Space group symmetry was used to generate the biological trimer that is seen in solution (Fig 3C).

**Table 3:** Structure alignment of human hCES1.

	RMSD (Å)		
hCES1	Wild type	S221A	N79Q
**Wild type**	-	0.44	0.47
**S221A**	-	-	0.30
**2h7c** *	0.50	0.48	0.48
**4ab1**	0.52	0.57	0.56
**1ya8** *	0.72	0.70	0.68

The structures were aligned using the PDBeFold server and the RMSD calculated between all Ca-atoms at best 3D superposition of the query and target structures (*average from all chains in the asymmetric unit).

Superimposition of the hCES1 structures onto examples of ligand-bound hCES1 complexes showed that ligand-binding does not change the overall conformational of the protein. In the case of the 2H7C, the root mean square deviation (rmsd) for all Cα atoms was 0.5 Å. (Table 3). The orientation of the side-chains of the catalytic triad (S221, E354, H467) (Fig 3D) in bound and unbound enzymes was also identical showing that the enzyme is pre-disposed for substrate binding.

Previously, it had been noted that trimer formation in hCES1 was mediated by two charge clamps across the trimer interface, from R186 and E183 of one monomer to E72 and K78 respectively, of the adjacent monomer [48]. Examination of the interfaces of the hCES1 structures presented here, and other structures of hCES1 (1mx1, 1yah, 2h7c), confirms that the E183 and K78 pairing does indeed form a salt bridge between two hCES1 monomer subunits molecules. However, the E72:R186 salt bridge was not apparent in the structures reported here, and in fact is only observed in the interface formed between monomer A and B of the trimer in PDB entry 1ya8. E72 takes up an alternate conformation in all other CES1 structures that have been reported. The acquisition of high resolution data, reported here, facilitated identification of a second salt bridge between residues K275 and E292. Examination of structures for which experimental data have been deposited with the PDB (2hrq, 3k9b, 1ya8, 1ya4 and 1yah) showed this interaction to be conserved. Therefore, we propose that the K275:E292 salt bridge together with the K78:E183 pairing, plays a potential role in stabilisation of the hCES1 trimer (Fig 3E).

## Conclusions

A comparison of authentically glycosylated human hCES1 produced in HEK cells with an aglycosylated version of the enzyme shows that preventing glycosylation at N79 does not affect the synthesis, activity or structure of the enzyme. In the hydrolysis of the model substrate 4-NPA, both hCES1 and aglycosylated hCES1 showed positive cooperatvity suggesting that substrate binding elicits conformation changes in the protein. However no major differences were observed in a comparison of crystal structures of hCES1 reported here and published ones with substrates/inhibitors bound into the active site. Further experiments comparing the solution behaviour of the proteins with/without substrates may be more revealing. More generally, the expression of hCES1 in mammalian cells has produced approximately fivefold higher yields than previously reported for insect cell expression. Crystal structures of hCES1 expressed in mammalian cells have been determined at </ = 2.0 Å, representing the highest resolution structures reported to date and provide crystal systems which could be exploited in the design of inhibitors which could be used to modulate the metabolism of clinically important drugs.
